# Not all that glittered is gold: neural mechanisms that determine when reward will enhance or impair memory

**DOI:** 10.3389/fnins.2014.00194

**Published:** 2014-07-16

**Authors:** David V. Clewett, Mara Mather

**Affiliations:** ^1^Neuroscience Graduate Program, University of Southern CaliforniaLos Angeles, CA, USA; ^2^Davis School of Gerontology, University of Southern CaliforniaLos Angeles, CA, USA; ^3^Psychology Department, University of Southern CaliforniaLos Angeles, CA, USA

**Keywords:** reward, context, norepinephrine, dopamine, memory

Rewards optimize future behavior by enhancing memory of events relevant to wellbeing. But how do rewards received in one context affect subsequent learning about the information previously associated with rewards? Updating existing knowledge is important to maximize rewards: when high-value stimuli are reinforced by reward in a new situation, contextual binding will support optimal decision-making later; however, contextual *un*binding can also improve wellbeing by preventing low utility associations from being formed.

Previous work indicates that initial emotional associations can impair later novel associations (Mather and Knight, 2008; Novak and Mather, 2009). To explore whether monetary rewards elicit memory impairments or enhancements, Madan et al. (2012) used a series of value-learning tasks to examine the effects of reward on explicit memory, implicit memory, and contextual binding. Consistent with earlier studies using emotional stimuli, high-value words were better recalled than low-value words on both implicit and explicit memory tests. Moreover, high-value words were more difficult to bind to a new temporal context, as measured by relatively later word output during free recall of a list. Interestingly, though free recall was equal for high- and low-value items, high-value words tended to proactively interfere with current list memory. These findings add to a growing literature showing that arousal can sometimes enhance and sometimes impair memory for context (see Mather and Sutherland, 2011), yet to date there is no neural model that can account for these dual effects. Below we discuss potential neural mechanisms that may help explain Madan et al.'s finding that rewards, much like other forms of arousal, impair subsequent context binding and proactively interfere with memory.

Motivationally significant events trigger release of the catecholamines, dopamine (DA) and norepinephrine (NE), which optimize central resource allocation to ensure that such events are remembered (McGaugh and Roozendaal, 2002; Adcock et al., 2006). However, how these brain systems sometimes lead to memory impairment is less understood. One possibility is that the mnemonic effect of arousal depends on the predictive value or goal-relevance of a salient item's context (Mather and Sutherland, 2011). Catecholamines are thought to broadcast prediction errors across the brain, providing a mechanism by which arousal can either enhance or impair memory (Hollerman and Schultz, 1998; Harley, 2004). Prediction errors arise from perceived discrepancies between expected and actual outcomes, and are therefore sensitive to changes in the context in which salient stimuli occur. In Madan et al.'s list-discrimination task, participants re-learned the high- and low-value words (along with novel words) in unrewarded lists. Thus, their finding that previous rewards impair rather than enhance memory binding to a new temporal context may reflect the influence of prediction errors on memory-updating processes driven by catecholamines.

The hippocampus represents associative information and selectively binds or unbinds contextual information in a manner consistent with prediction errors (Mizumori, 2013). Thus, in the study by Madan et al. (2012), reduced DA in the hippocampus may have impaired contextual binding due to a lack of reward reinforcement during re-learning (i.e., negative prediction error). Contextual mismatches also engage the orbitofrontal cortex (OFC), which helps update memory representations to account for shifting reward contingencies, such as during reversal learning (O'Doherty et al., 2001; Rolls, 2004). Similarly, in monkeys, phasic NE release signals changes in stimulus-reward associations (Aston-Jones et al., 1997), a process that is likely driven by context-specific inputs from the OFC (Aston-Jones and Cohen, 2005). Taken together, these findings suggest that arousal's influence on associative memory may be determined by catecholaminergic modulation of brain structures that track the predictive value of contextual information.

An interesting implication of this prediction error account is that punishment-related incentives should enhance associative binding during the list-discrimination task. Updating previously threatening stimuli to represent a safer environment would support memory of cues that also promote wellbeing. However, this account contradicts the prevailing view that arousal-induced memory impairments are invariant to stimulus valence (Mather and Knight, 2008). Thus, one alternative explanation is that arousal's selective influence on binding is not determined by valence but rather by the priority of the context in which an arousing item is encoded.

According to the *arousal-biased competition* (ABC) theory, arousal will amplify the effects of priority such that memory of goal-relevant stimuli is enhanced, while memory of less important information is suppressed (Mather and Sutherland, 2011). Since there was no instruction to pay special attention to the new lists in Madan et al.'s study, any arousal induced by remembering a prior reward would impair context binding. However, if the experimenters explicitly directed participants to prioritize learning the current list context, ABC theory would predict that arousal should enhance rather than impair binding of high-value words to their current list. Consistent with this account, one recent study demonstrated that harbinger cues that predict emotional stimuli enhance rather than impair associative binding when participants are made aware of their contingencies (Sakaki et al., 2014). We propose that catecholamines also play a key role in ABC, since they mediate arousal's selective influence on cognitive processing (Robbins and Arnsten, 2009).

In summary, Madan et al.'s findings underscore that adaptive behavior not only relies on the ability of rewards to enhance memory of significant stimuli but to also impair binding of new contextual associations that do not predict such events. We propose that DA and NE help determine reward's divergent effects on memory by interacting with regions that update the salience and priority of a stimulus. Whereas such neuromodulation optimizes wellbeing under normal circumstances, acute stress appears to hijack these mechanisms in ways harmful to behavior. Acute stress amplifies reward salience (Mather and Lighthall, 2012), resulting in a shift from goal-directed processes toward habitual responding (Schwabe and Wolf, 2011). This stress-induced shift appears to be driven by elevated levels of catecholamines and glucocorticoids in the OFC (Robbins and Arnsten, 2009), which reduce its sensitivity to reward devaluation (van Eimeren et al., 2009; Schwabe et al., 2011, 2012). Under normal circumstances, the updating of emotional but not neutral associations relies on greater OFC activation (Sakaki et al., 2011; Nashiro et al., 2012, 2013). Thus, the impairing effects of elevated stress hormones on OFC function may be one mechanism by which devalued items proactively interfere with memory. Ultimately, understanding how arousal affects associative memory has important implications for other pathologies, such as post-traumatic stress disorder, in which the inability to restrict fear to the appropriate context leads to intrusive thoughts and impairs day-to-day life.

## Conflict of interest statement

The authors declare that the research was conducted in the absence of any commercial or financial relationships that could be construed as a potential conflict of interest.
